# The occurrence of *Culicoides* species, the vectors of arboviruses, at selected trap sites in Zimbabwe

**DOI:** 10.4102/ojvr.v82i1.900

**Published:** 2015-05-29

**Authors:** Stuart J.G. Gordon, Charlotte Bolwell, Chris Rogers, Godfrey Musuka, Patrick Kelly, Karien Labuschagne, Alan J. Guthrie, Eric Denison, Philip S. Mellor, Christopher Hamblin

**Affiliations:** 1 Institute of Veterinary, Animal and Biomedical Sciences, Massey University, New Zealand; 2 Polio Communication Section, UNICEF Nigeria Country Office, Nigeria; 3 Ross University School of Veterinary Medicine, St. Kitts, West Indies; 4 ARC-Onderstepoort Veterinary Institute: Parasites, Vectors and Vector-borne Diseases, Onderstepoort, South Africa; 5 Equine Research Centre, University of Pretoria, South Africa; 6 The Pirbright Institute, Pirbright, United Kingdom

## Abstract

A study of the distribution of *Culicoides* species was conducted by establishing 12 light trap sites over five rainy seasons between 1998 and 2003 covering all the geo-climatic natural regions of Zimbabwe. In total, 279 919 specimens of *Culicoides* were trapped over a total of 163 trapping nights. The highest median counts of *Culicoides* per trapping night were recorded in natural region III, which has climatic conditions conducive to the successful development of the larvae. *Culicoides imicola*, the major vector of bluetongue and African horse sickness viruses in Africa, was found to be the most abundant species (80.4%), followed by *Culicoides enderleini* (5.9%) and *Culicoides milnei* (5.2%). This study identified 10 species of *Culicoides* that had not been previously described in Zimbabwe, including *Culicoides loxodontis* and *Culicoides miombo,* which are members of the *C. imicola* complex. A total of 23 994 *Culicoides* midges were collected from five trap sites in Harare, Zimbabwe, with the dominant species, *C. imicola,* representing 91.6% of the total collection. Seventeen arboviruses were isolated from these midges, 15 of which were bluetongue virus. The predominant bluetongue virus serotype was serotype 11, followed by serotypes 1, 8, 12 and 15. Bluetongue virus serotypes 1, 2, 8, 10, 12, 15, 16 and 18, detected in this study, had not been previously reported in Zimbabwe.

## Introduction

African horse sickness virus (AHSV), which infects all equine species, and bluetongue virus (BTV), which infects sheep and other ruminants, are endemic in Zimbabwe. High seroprevalences of AHSV in horses and donkeys and BTV in cattle and sheep were reported throughout Zimbabwe (Gordon 2010), with mortality rates because of African horse sickness and bluetongue reaching 95% (Coetzer & Guthrie 2004) and 33% (Musuka 1999) respectively. Based on their economic importance and capacity to spread rapidly between countries, the World Organisation for Animal Health (OIE) has designated both these diseases as notifiable animal diseases. Equine encephalosis virus (EEV), which infects all equine species, and epizootic haemorrhagic disease virus (EHDV), which infects domestic and wild ruminants, have also been detected in Zimbabwe (Gordon 2010; Musuka 1999; Paweska *et al.* 1999). Many of these viruses are also enzootic in neighbouring countries, namely Botswana (Mushi *et al.* 1998), Zambia (Mweene *et al.* 1996), Namibia and South Africa (Meiswinkel, Venter & Nevill 2004; Scacchia *et al.* 2009; World Organisation for Animal Health 2011). Oura *et al.* (2012) recently documented EEV in Ethiopia, Ghana, Gambia and Israel. Bluetongue today has a nearly worldwide distribution.

All of these diseases are caused by double-stranded ribonucleic acid (dsRNA) viruses of the family *Reoviridae* and genus *Orbivirus*, which are transmitted almost exclusively by *Culicoides* (Diptera: Ceratopogonidae) biting midges (Meiswinkel *et al.* 2004; Mellor, Boorman & Baylis 2000). *Culicoides imicola* and *Culicoides bolitinos* have been identified as vectors of AHSV and are widely distributed in sub-Saharan Africa (Meiswinkel & Paweska 2003; Mellor 1993). *Culicoides bolitinos* has also been implicated as an important potential vector of EEV and BTV in South Africa (Meiswinkel & Paweska 1998, 2003; Venter *et al.* 2002). 

Previous studies in Zimbabwe found *C. imicola* to be the most abundant livestock-associated *Culicoides* species, followed by *Culicoides zuluensis* (Blackburn, Searle & Phelps 1985; Braverman & Phelps 1981; Braverman *et al.* 1985; Gordon 2010; Musuka 1999; Musuka, Chihota & Kelly 1998; Phelps, Blackburn & Searle 1982). The vector competence of most *Culicoides* species in Zimbabwe has not been studied in detail and other species may, in future, be identified as vectors of these viruses.

Agro-ecological classification, known as natural region (NR) classification in Zimbabwe, divides the country into five regions based on mean annual rainfall (Vincent & Thomas 1960). The aim of this study was to identify the species of *Culicoides* distributed across these regions during the period 1998–2003. It is known that climate has a profound influence on the viability and fecundity of the *Culicoides* midges (Mellor 1993). To date no differences in disease abundance have been reported between these regions. The monthly abundance and distribution of *Culicoides* species in these five regions was, therefore, determined. In addition, this study aimed to isolate and identify arboviruses isolated from *Culicoides* midges collected in Harare over 7 days within the study period. Such studies will expand our knowledge of the biology of Zimbabwean *Culicoides* species and the potential for transmission of viral pathogens. This information will help assess the risks to both ruminant and equine livestock in Zimbabwe attributable to the presence of the *Culicoides* vector and hence the implementation of appropriate integrated control strategies (Rawlings *et al.* 1998).

## Materials and methods 

### Selection of *Culicoides* trap sites in Zimbabwe

**FIGURE 1:**
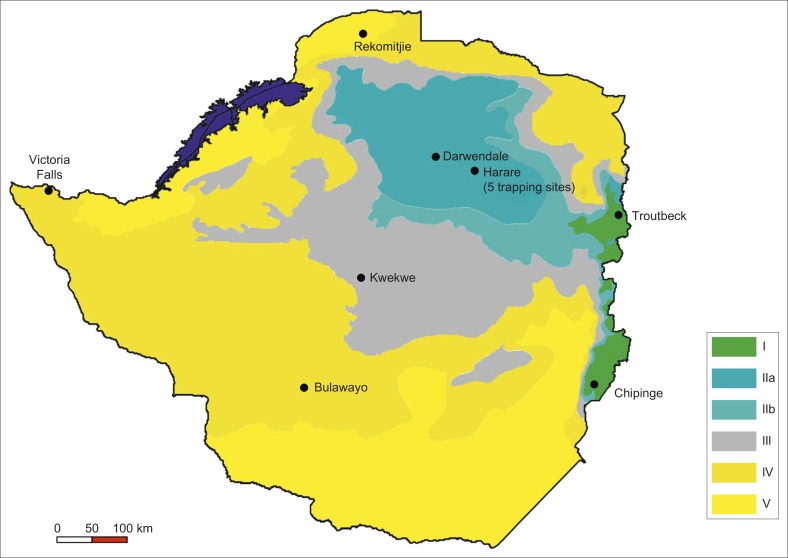
The natural regions of Zimbabwe and the locations of the 12 *Culicoides* trapping sites.

*Culicoides* midges were collected from a convenience sample of 12 trap sites in Zimbabwe. Collections were made over five consecutive rainy seasons (November–April) from 1998 to 2003. Collection site selection was based on several criteria, including: a minimum number of either horses or ruminants (> 8); historical presence of clinical disease or deaths in animals; presence of suitable *Culicoides* vector breeding conditions (e.g. low-lying areas, such as river banks or marshy fields, with clay soils); security of the equipment; and the availability of an alternating 220V current power supply. Trap sites were distributed to include each of the five NRs of Zimbabwe (Figure 1). Two trapping sites were established in NR I, six in NR II, one in NR III, two in NR IV and one in NR V. Twenty-four individual collections (trapping nights) were made from NR I, 109 from NR II, 18 from NR III and 9 from NR IV across the five rainy seasons. Trapping was to be conducted for one night every 2 weeks during each rainy season, although this protocol was not followed consistently at each trapping site. Only three individual collections were made in NR V during the entire period under study.

Rainfall is highest in NR I (> 1050 mm per annum) whilst NR II and III contain the most arable land and receive 500 mm – 1050 mm of rain per annum. NR I is a specialised and diversified farming region with plantation forestry, fruit and intensive livestock production, whilst NRs II and III are suitable for intensive farming based on crops and livestock production. Severe mid-season dry spells make NR III marginal for maize, tobacco and cotton, or for enterprises based on crop production alone. The farming systems, therefore, are based on both livestock (assisted by the production of fodder crops) and cash crops. NR IV and V have a lower annual rainfall (< 600 mm per annum) and experience periodic seasonal droughts and/or severe dry spells during the rainy season. The farming, therefore, is based on extensive livestock ranching and drought-resistant fodder crops (Vincent & Thomas 1960). 

### Trapping, sorting and processing of *Culicoides* samples for identification

Dusk (18:00) till dawn (06:00) trapping was conducted at each trap site using Onderstepoort 220 V down draught suction light traps each fitted with an 8 W black fluorescent (ultraviolet) light tube. The traps were hung on the branches of trees or on the eaves of buildings approximately 1.4 m above ground level and within close proximity to the animals. *Culicoides* midges were collected into phosphate-buffered saline (PBS) and 0.5% chlorhexidine (Savlon^®^, Novartis, U.K.). After collection specimens were re-suspended and preserved in 80% ethyl alcohol and stored in the dark at room temperature until sorted, identified and counted.

Species were identified and counted under a binocular dissecting microscope using a wing pattern atlas of southern African *Culicoides* species (R. Meiswinkel, unpublished atlas 1996). For large collections in excess of 1000 midges, the total numbers were estimated by measurement of a minimum of five 1 mL subsamples according to the protocol of Van Ark and Meiswinkel (1992).

### Trapping and sorting of *Culicoides* midges in Harare for virus isolation

*Culicoides* midges were collected for virus isolation at five sites around Harare (17°51′50′S, 31°1′47′E, located in NR II) using the method described above. These five Harare sites formed part of the 12 trappings mentioned previously. These *Culicoides* species were age graded according to the method described by Dyce (1969). Harare is 1508 m above sea level and situated in the northeast highveld of Zimbabwe. It is characterised by moderate subtropical conditions with an average monthly rainfall of 120 mm and air temperature between 15 ºC and 27 ºC. All trap sites were on smallholdings with vertisol soil types and mixed forest vegetation where urban-intensive farming was practised. The main species of livestock were cattle, goats, sheep and horses.

The traps were hung from branches or eaves of buildings within close proximity to animal premises. All sites’ traps were operated from dusk (18:00) to dawn (06:00) for seven consecutive days in March 2003.* Culicoides* were collected into a phosphate-buffered saline (PBS) solution containing 100 mg/mL of gentamicin and 0.1% chlorhexidine (Savlon^®^, Novartis, U.K.) and stored at 4 ºC until processed. 

Individual species of *Culicoides* were stored at 4 ºC in 2 mL Eppendorf tubes (a maximum of 50 flies per Eppendorf tube) with 1 mL of PBS containing 100 mg/mL of gentamicin and 0.1% chlorhexidine (Savlon^®^, Novartis, U.K.). For each species or species group, the *Culicoides* specimens were sorted into the following categories: males, blood engorged females, non-engorged nulliparous females and non-engorged parous females, according to Dyce (1969). 

### Preparation of *Culicoides* specimens for virus isolation and identification

Midges were grouped into individual pools of up to 100 non-engorged females of each species. Each pool was homogenised in a mortar using a pestle with 1 mL of Eagles maintenance medium containing 1% foetal calf serum and antibiotics, using a modification of the technique proposed by El Hussein *et al.* (1989). Each homogenate was subsequently titrated for infectious virus in BHK-21 cells, brains from suckling mice and 11-day-old embryonated hens’ eggs. Virus isolates were characterised using serogroup-specific, indirect, sandwich enzyme-linked immunosorbent assay (ELISA) for BTV (Thevasagayam *et al.* 1996), AHSV (Hamblin, Mellor & Boned 1991), EEV (Crafford *et al.* 2003) and EHDV (Thevasagayam *et al.* 1996) antigens. The BTV isolates were subsequently serotyped using virus neutralisation index assays (Hazrati & Ozawa 1965). 

## Results

### Collection of *Culicoides* across Zimbabwe for species identification

A total of 279 919 specimens of *Culicoides,* representing 51 different species, were trapped across Zimbabwe over five rainy seasons. Table 1 shows the numbers of *Culicoides* trapped per night in each NR of Zimbabwe and are reported as median values with interquartile ranges (IQRs). The distribution of each of the 10 most abundant *Culicoides* within the NR are presented as percentage values in Table 2. The trap sites located in NR III recorded the highest median count of *Culicoides* species (1376 [IQR 286–1980]), followed by those trap sites located in NR IV (821 [IQR 175–918]). A full list of the different *Culicoides* trapped (in order of abundance) across the five NRs of Zimbabwe between 1998 and 2003 is shown as supplementary data in Appendix 1 (Table 1-A1).

**TABLE 1:** Median (and interquartile ranges) counts of *Culicoides* midges per trapping night caught in the five natural regions of Zimbabwe across five rainy seasons: 1998–2003 (November–April).

Number	Number of established trapping sites	Total number of individual collections (trapping nights)	Median (IQR) counts of *Culicoides* per trapping night	Minimum number of *Culicoides* per trapping night	Maximum number of *Culicoides* per trapping night
I	2	24	5 (1–25)	0	1320
II	6	109	125 (37–678)	0	10 473
III	1	18	1376 (286–1980)	9	4026
IV	2	9	821 (175–918)	56	8037
V	1	3	255 (148–3461)	148	3461

NR, natural region; IQR, interquartile range.

**TABLE 2:** The mean percentage of the 10 most abundant *Culicoides* spp. trapped in each natural region in Zimbabwe over the five rainy seasons (1998–2003).

*Culicoides* species	Natural region (%)					
	I	II	III	IV	V	Total
*C. imicola*	84.7	80.2	81.7	85.0	2.7	80.4
*C. enderleini*	2.3	2.5	6.2	5.3	75.6	5.9
*C. milnei*	0.8	14.2	1.2	0.7	0.0	5.2
*C. zuluensis*	2.6	1.4	7.1	0.0	0.0	4.8
*C. nevilli*	0.0	0.2	1.8	0.03	0.0	1.2
*C. leucostictus*	0.04	0.2	0.5	0.06	0.0	0.4
*C. miombo*	3.9	0.03	0.0	5.4	3.4	0.4
*C. pycnostictus*	0.04	0.4	0.3	0.0	0.0	0.3
*C. bolitinos*	0.1	0.2	0.2	0.2	5.7	0.3
*C. subschultzei*	0.0	0.03	0.2	0.9	0.5	0.2

*C*., *Culicoides*.

The predominant *Culicoides* species trapped was *C. imicola* (80.4%), followed by *Culicoides enderleini* (5.9%), *Culicoides milnei* (5.2%) and *C. zuluensis* (4.8%). *Culicoides imicola* was the most abundant species trapped in NR I–IV, with *C. enderleini* being the most abundant species trapped in NR V. The second most abundant species trapped in each NR was *Culicoides miombo* (NR I), *C. milnei* (NR II), *C. zuluensis* (NR III), *C. miombo* (NR IV) and *C. bolitinos* (NR V). Species of *Culicoides* found in every NR were *C. imicola, C. enderleini, C. bolitinos* and *Culicoides subschultzei*. Ten additional species, which have not previously been recorded in Zimbabwe, were collected (Table 3), bringing the total number of *Culicoides* species recognised in Zimbabwe to 60.

**TABLE 3:** The distribution of previously unreported species of *Culicoides* across the five natural regions of Zimbabwe (1998–2003).

***Culicoides* spp.**	**NR**				
	I	II	III	IV	V
*C. *sp. # 107(provisionally given the name *C. kwagga *by Meiswinkel [1995] but not officially described)	-	√	-	√	-
*C. glabripennis*	-	√	-	-	√
*C. kerichoensis*	√	-	-	-	-
*C. loxodontis*	-	√	-	√	√
*C. miombo*	√	√	-	√	√
*C. moreli*	-	-	-	√	-
nr. *angolensis *(species closely associated with* C. angolensis*)	√	-	-	-	-
*C. ovalis*	√	-	-	-	-
*C. shimoniensis*	-	-	√	-	-
*C. trifasciellus*	√	√	-	-	√

*C*., *Culicoides;* NR, natural region.

### Collection of *Culicoides* in Harare for species identification and virus isolation

A total of 23 994 *Culicoides* specimens were collected from the Harare sites. *Culicoides imicola* (21 986/23 994; 91.6%) was the most prevalent of the 26 species captured, followed by *C. enderleini* (1556/23 994; 6.5%). Other species of *Culicoides* regularly captured at Harare were *C. zuluensis* (248/23 994; 1.0%), *C. bolitinos* (70/23 994; 0.3%) and *Culicoides brucei* (39/23 994; 0.2%). Three species novel to Zimbabwe, namely *C. miombo*, *Culicoides glabripennis* and a specimen from the Nigripennis group, were identified in the Harare collections. Significant differences were found in the numbers of *Culicoides* midges collected at the five Harare trapping sites. Nearly 90% of all the specimens collected were from eight collections made at one of the sites at a horse stud. However, the greatest species diversity was observed at a different horse stud (17 different species). The lowest numbers of *Culicoides* and lowest species diversity were found at a dairy farm on the outskirts of Harare that had cattle only and no horses (196 midges belonging to eight species of *Culicoides*).

In the Harare study, 222 samples of non-engorged parous female *Culicoides* were assayed for BTV, AHSV, EEV and EHDV. A total of 17 *Orbiviruses* were isolated: 15 BTV and 2 EEV. Fourteen isolates of BTV were made from *C. imicola* and one from *Culicoides magnus.* Both the EEV isolates were from *C. imicola*. No AHSV or EHDV were isolated. Nine different BTVs were identified. Three isolates of serotype 11 (3/15; 20%) were made and two isolates each of serotypes 1, 8, 12 and 15 (2/15; 13.3% each) were made. One isolate each of serotypes 2, 10, 16 and 18 were also identified (1/15; 6.7% each). The EEV isolates were not serotyped.

## Discussion

Trap sites chosen were believed to be representative of the climatic and geo-physical conditions found within each NR. However, the significance of the distribution of *Culicoides* across the five NRs of Zimbabwe must be interpreted with caution. It is important to realise that these five agro-ecological regions were characterised more than 50 years ago (Vincent & Thomas 1960). Mugandani *et al.* (2012) have argued that this is misleading as it implies that the climatic conditions have remained stable over this time. Research, however, points to the contrary; Makarau (1999) and Low (2005) noted increased variability of rainfall, rain days and temperature in Zimbabwe. Most meteorological stations in Zimbabwe have shown a decline in rainfall over the past 100 years (Mugandani *et al.* 2012). Furthermore, Corbett and Carter (1996) noted that continued use of these agro-ecological regions was at odds with the political, social and agrarian reform that has taken place in Zimbabwe since Vincent and Thomas (1960) established these regions. 

Caution should also be observed when interpreting absolute counts of *Culicoides* as there may be some variation dependent on the trap sites within each NR. The number of host animals and the distance of these animals from the trap site may also influence the numbers of *Culicoides* collected. Trap sites in NR II recorded the greatest diversity of *Culicoides* species, with 43 species identified. The highest median count of *Culicoides* was recorded in trap sites located within NR III. Natural region III includes the central highveld of Zimbabwe, averaging a total annual rainfall of 500 mm – 700 mm, with predominantly greyish brown clay soils and sandy loams derived from granitic rocks (Vincent & Thomas 1960). High median counts of *Culicoides* midges were recorded in March and April, at the end of the rainy season. Numbers of adult flies steadily increase after the start of the rainy season as the availability of breeding sites increases (Musuka 1999). Similar findings have been reported previously in Zimbabwe and South Africa, with large populations of *Culicoides* vectors found in areas with high average annual rainfall and summer temperatures (Musuka 1999; Musuka *et al.* 2001; Venter, Nevill & Van der Linde 1997). Temperature influences the abundance of *Culicoides* vectors through its effect on the availability of suitable breeding sites, larval development and adult mortality (Mellor & Hamblin 2004). High environmental temperatures lead to a shorter time span between blood-feeding events and an increase in the rate of virogenesis within the adult vector, leading to earlier virus transmission, although at high temperatures the rate of adult mortality also increases (Mellor & Hamblin 2004; Wellby *et al.* 1996).

Previous studies have demonstrated that soil type appears to be very important in determining the distribution and abundance of *C. imicola* (Meiswinkel 1998; Musuka *et al.* 2001). In concurrence with these previous findings, the largest numbers of *C.* *imicola* were found in areas with a high, moisture-retentive clay soil, whilst the lowest numbers were encountered in rapidly draining sandy soils. The biggest collections of *Culicoides* were obtained from a large equine stud on the outskirts of Harare. This farm was situated in a low-lying area on the banks of a major river and had clayey soils and open grassland vegetation. 

This study showed that large numbers of *C. imicola* were collected in light traps located at sites where horses were stabled indoors at night even though some stables were fitted with protective fly screens. This suggests that *C. imicola* were still feeding successfully as they depend upon blood meals for the development of their eggs. Successful feeding may have been facilitated by the common practice of leaving the upper half of the stable door open for ventilation (Meiswinkel 1998). Horses kept out in the open at night appear to be more susceptible to midge bites than stabled horses and these outside horses may act as decoys deflecting *C. imicola* away from the stabled animals. If these decoys are removed or stabled, then the midges may attempt to feed on stabled animals (Meiswinkel 1998). 

This study has identified 10 species of *Culicoides* that previously have not been recorded in Zimbabwe (Blackburn *et al.* 1985; Musuka 1999; Musuka & Kelly 2000; Musuka *et al.* 1998, 2001; Phelps *et al.* 1982), bringing the total number of recognised species of *Culicoides* in Zimbabwe to 60. Of particular note was the identification of two additional *C. imicola* complex species, *Culicoides loxodontis* and *C. miombo*, which were distributed across four of the NRs of Zimbabwe. The epidemiological significance of these species as vectors of arboviruses in Zimbabwe needs further investigation, although the findings of this study significantly add to the available knowledge on the distribution of these species in southern Africa.

Musuka (1999) has shown that *C.* *bolitinos* has a widespread distribution in southern Africa. Furthermore, *C. bolitinos*, which was found at all five trap sites in Harare in the present survey, has been implicated in AHSV, EEV and BTV transmission in South Africa (Meiswinkel & Paweska 1998, 2003; Venter *et al.* 2002) and, therefore, could play a role in the spread of the disease in Zimbabwe. In our study, the highest prevalence (5.7%) of *C. bolitinos* was recorded in NR V (Table 2). This region is characterised by low rainfall and high environmental temperatures, although in South Africa *C. bolitinos* has been reported to be more abundant in the cooler mountainous regions (Meiswinkel & Paweska 2003; Venter *et al.* 2002). This species breeds in the dung of cattle and buffaloes and the traps in the NR V trap site were located near a cattle pen containing 30 cattle. The trap sites also included a wildlife sanctuary in north Zimbabwe that hosts large populations of wild buffaloes (*Syncerus caffer*).

The distribution of *Culicoides* species observed during this study closely matched the sero-incidence and prevalence of AHSV, EEV, BTV and EHDV reported by Gordon (2010). In order to fully understand the role of the various *Culicoides* species in the transmission of arboviruses in Zimbabwe, further studies are needed to determine the vector competence of the sibling species of the *C. imicola* group and other *Culicoides* species that are potential vectors in southern Africa (Musuka *et al.* 1998). It has recently been demonstrated, for example, that *C. magnus* could become infected with both BTV and AHSV under laboratory conditions (Paweska, Prinsloo & Venter 2003; Paweska, Venter & Mellor 2002; Venter *et al.* 2004, 2009).

In this study, virus isolations of EEV and BTV were made only from *C. imicola* and *C. magnus*. According to Meiswinkel *et al.* (2004), *C*. *miombo* is suspected to be a vector of BTV. In the Harare study, however, no viral isolates were obtained from this species (although only nine individual specimens were collected and assayed). No AHSV or EHDV were isolated from any of the trapped *Culicoides* species despite the traps being located at horse studs and cattle farms. Low AHSV isolations from *Culicoides* midges were also reported by Rawlings *et al.* (1998) in a study conducted in Gambia, which was attributed to an extremely low infection prevalence of AHSV in the vector species of *Culicoides*. These observations were further authenticated by Mellor, Osborne and Jennings (1984), in Sudan, who could only make two AHSV isolations out of 7000 *Culicoides* specimens. Many viral, genetic and environmental factors can influence the susceptibility of *Culicoides* to infection and some species may be refractory to infection (Mellor *et al.* 1984). It is recommended, however, that large numbers of flies from many different collection sites should be collected and assayed before defining a species as refractory to infection.

The predominant serotype of BTV isolated from the *Culicoides* vectors was serotype 11, followed by serotypes 1, 8, 12 and 15. Previously only BTV serotype 11 has been isolated from the *Culicoides* vector in Zimbabwe (Blackburn *et al.* 1985), so this is the first time that BTV serotypes 1, 2, 8, 10, 12, 15, 16 and 18 have been recorded in the country. The possibility that some of these additional BTV serotypes isolated may be vaccine viruses should be considered, however. The vaccine currently used in Zimbabwe is an Onderstepoort Biological Product^®^ attenuated cell culture adapted vaccine. Serotypes 15, 16 and 18 are not included in this vaccine and thus vaccinated cattle and sheep may not be adequately protected against all BTV serotypes present in Zimbabwe. There is, therefore, a need to consider the possibility of developing a local polyvalent vaccine that protects against all the BTV serotypes circulating in Zimbabwe.

## Conclusion

The greatest diversity and highest median counts of *Culicoides* were found in the central highveld region of Zimbabwe, which enjoys climatic and geophysical conditions favouring optimal vector breeding and larval development. The discovery of new *Culicoides* species in Zimbabwe adds to the existing knowledge on the distribution of *Culicoides* species in southern Africa. These findings, however, highlight the need for further studies to measure the vector competence of existing* Culicoides* species and to fully understand the role of these species in the transmission and epidemiology of arboviruses in Zimbabwe.

Furthermore, the discovery of novel BTV serotypes in this study highlights the need to review bluetongue vaccines currently in use and vaccination and control strategies being practised in Zimbabwe.

## Appendix 1

**TABLE 1-A1:** The distribution of all species of *Culicoides* collected across the five natural regions of Zimbabwe (1998–2003).

*Culicoides* species	NR				
	I	II	III	IV	V
*imicola*	√	√	√	√	√
*enderleini*	√	√	√	√	√
*milnei*	√	√	√	√	
*zuluensis*	√	√	√		
*nevilli*		√	√	√	
*leucostictus*	√	√	√	√	
*miombo*	√	√		√	√
*pycnostictus*	√	√	√		
*bolitinos*	√	√	√	√	√
*subschultzei*		√	√	√	√
*coarctatus*		√	√		
*tropicalis*	√	√	√	√	
*loxodontis*				√	√
*brucei*	√	√	√		
*nivosus*	√	√	√	√	
*magnus*		√	√		
*similis*		√	√	√	
*trifasciellus*	√	√			
*engubandei*	√	√	√	√	
*tororoensis*				√	√
Nigripennis group		√	√	√	√
sp. #54 (p/f)				√	
sp. #90		√		√	√
*neavei*	√	√	√	√	
*ravus*		√	√	√	√
*gulbenkiani*	√	√			
*schultzei*			√	√	
*moreli*				√	
sp. #50		√		√	√
*exspectator*		√	√	√	√
*albopunctatus*		√	√		
sp. #107		√		√	
*tuttifrutti *		√		√	
sp. #54 (d/f)				√	√
*kanagai*		√		√	
*ovalis*	√				
sp. #69			√		
*glabripennis*					√
*fulvithorax*		√	√		
*cornutus*		√			
*moucheti*				√	
*kibatiensis*	√	√			
*perettii*		√		√	
sp. #94		√			
*dutoiti*			√	√	
nr. angolensis	√				
*krameri*	√	√			
*shimoniensis*			√		
nr. glabripennis sp. #7		√			
*dekeyseri*		√			
Accraensis group			√		

NR, natural region.
